# Effect of irradiation on the survival and susceptibility of female *Anopheles arabiensis* to natural isolates of *Plasmodium falciparum*

**DOI:** 10.1186/s13071-020-04135-w

**Published:** 2020-05-20

**Authors:** Edwige Guissou, Serge Poda, Domombabele François de Sales Hien, Serge Rakiswende Yerbanga, Dari Frédéric Da, Anna Cohuet, Florence Fournet, Olivier Roux, Hamidou Maiga, Abdoulaye Diabaté, Jeremie Gilles, Jérémy Bouyer, Anicet G. Ouédraogo, Jean-Baptiste Rayaissé, Thierry Lefèvre, Kounbobr Roch Dabiré

**Affiliations:** 1 Institut de Recherche en Sciences de la Santé, Bobo-Dioulasso, Burkina Faso; 2 MIVEGEC, Montpellier University, IRD, CNRS, Montpellier, France; 3 Laboratoire mixte international sur les vecteurs (LAMIVECT), Bobo Dioulasso, Burkina Faso; 4 Université Nazi Boni, Bobo Dioulasso, Burkina Faso; 5 Insect Pest Control Laboratory, Joint FAO/IAEA Division of Nuclear Techniques in Food and Agriculture, Vienna, Austria; 6 Centre International de Recherche-Développement sur l’Elevage en zone Subhumide, Bobo-Dioulasso, Burkina Faso; 7 Centre de Recherche en Écologie et Évolution de la Santé (CREES), Montpellier, France

**Keywords:** Sterile insect technique (SIT), Competence, Direct membrane feeding assay

## Abstract

**Background:**

The sterile insect technique (SIT) is a vector control strategy relying on the mass release of sterile males into wild vector populations. Current sex separation techniques are not fully efficient and could lead to the release of a small proportion of females. It is therefore important to evaluate the effect of irradiation on the ability of released females to transmit pathogens. This study aimed to assess the effect of irradiation on the survival and competence of *Anopheles arabiensis* females for *Plasmodium falciparum* in laboratory conditions.

**Methods:**

Pupae were irradiated at 95 Gy of gamma-rays, and emerging females were challenged with one of 14 natural isolates of *P. falciparum*. Seven days post-blood meal (dpbm), irradiated and unirradiated-control females were dissected to assess the presence of oocysts, using 8 parasite isolates. On 14 dpbm, sporozoite dissemination in the head/thorax was also examined, using 10 parasites isolates including 4 in common with the 7 dpbm dissection (oocyst data). The survivorship of irradiated and unirradiated-control mosquitoes was monitored.

**Results:**

Overall, irradiation reduced the proportion of mosquitoes infected with the oocyst stages by 17% but this effect was highly inconsistent among parasite isolates. Secondly, there was no significant effect of irradiation on the number of developing oocysts. Thirdly, there was no significant difference in both the sporozoite infection rate and load between the irradiated and unirradiated-control mosquitoes. Fourthly, irradiation had varying effects on female survival with either a negative effect or no effect.

**Conclusions:**

The effect of irradiation on mosquito competence strongly varied among parasite isolates. Because of such isolate variability and, the fact that different parasite isolates were used to collect oocyst and sporozoite data, the irradiation-mediated reduction of oocyst prevalence was not confirmed for the sporozoite stages. Our data indicate that irradiated female *An. arabiensis* could contribute to malaria transmission, and highlight the need for perfect sexing tools, which would prevent the release of females as part of SIT programmes.
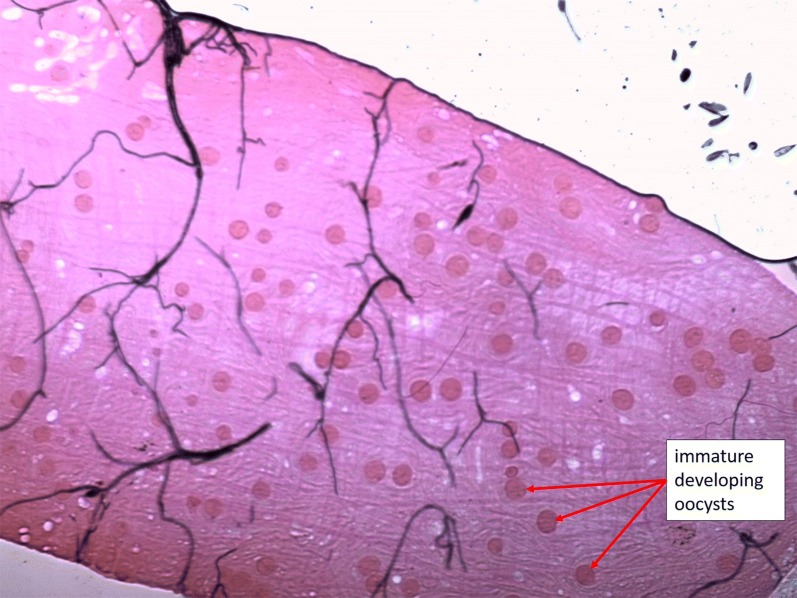

## Background

The worldwide annual incidence of malaria declined by 36% between 2000 and 2015 [1]. Control measures based on vector management have played an important role in reducing malaria transmission with, for example, the use of long-lasting insecticide-treated nets contributing to an estimated 68% of the decline in *Plasmodium falciparum* incidence over this period [2]. Since 2015 however, global progress has stalled, and several African countries are currently experiencing an increase in malaria incidence [3]. The reasons for these recent increases are unclear but current vector control techniques are showing some limitations. This may include a loss of motivation in tool use [4], and/or vector adaptations such as physiological and behavioral resistance to insecticides [5, 6].

Although improving the use of existing and available tools is essential for malaria control in the near future, there is also an urgent need for the development and implementation of alternative solutions [7]. One solution is based on the sterile insect technique (SIT), which aims to control vector populations by releasing sterile males. SIT relies on the massive production of sterile males by irradiation or chemical treatment and introduction into wild insect populations in order to reduce the number of adults in subsequent generations [8–10]. With repeated release, this approach has proven successful in eliminating some agricultural pest species [11], and has shown to be promising in suppressing or reducing the density of disease vectors from islands [12] or from relatively isolated areas such as urban settings [13]. More recently, it allowed the elimination of two partially isolated populations of *Aedes albopictus* in Guangzhou, China, when used in combination with the incompatible insect technique [14].

In recent years, the joint FAO/IAEA programme has spurred renewed interest in the development of SIT approaches for the control of mosquito-borne diseases [15, 16]. With regard to malaria, *Anopheles arabiensis* has focused much of the scientific attention as this species can display localized, narrow range distribution such as river side [17] or urban areas [18, 19]. Accordingly, the radiation biology of this species has been relatively well studied [20–25]. Besides efficient mass-rearing and optimal level of irradiation ensuring male sterilization with limited impact on sexual competitiveness, a perfect separation technique of male and female mosquitoes prior to release is essential [26].

To date, the available sexing tools, including pupal size, addition of toxicants to blood-meal sources, or development of genetic sexing strains, remain imperfect; and a small proportion of females can escape sexing before irradiation [21, 26–28]. These females will be irradiated with the males and can therefore potentially contribute to malaria transmission when released into wild populations. While efforts to find an effective and operational sex separation technique are maintained, it is important to evaluate the effect of irradiation on the ability of female *Anopheles* to transmit *P. falciparum*. Previous work has focused on the influence of irradiation on a large range of traits including sperm production [23, 29, 30], male competitiveness [23, 29, 31], male and female longevity [20, 28], insemination rate [32], oviposition behavior [32] and fertility and fecundity [20, 32], but no study has, to our knowledge, characterized the influence of irradiation on the competence of *An. arabiensis* for *P. falciparum*.

Competence, the mosquito’s ability to ensure parasite development and transmission, is a combined estimate of parasite infectivity and vector susceptibility to infection. It thus encompasses both mosquito resistance mechanisms used to fight the infection and parasite mechanisms used to overcome the vector’s defenses [33]. The molecular and genetic bases of mosquito competence for malaria parasites have been well characterized for a number of mosquito-parasite associations [34, 35], and there is also great diversity of ways in which biotic and abiotic environmental factors (temperature, mosquito diet, insecticide exposure, microbial gut flora, etc.) can affect mosquito competence [36]. As any other environmental factor, radiation also has the potential to influence the competence of *Anopheles* vectors for *P. falciparum*. For example, *Aedes aegypti* mosquitoes exposed to a 5000 roentgen dose of X ray-irradiation and infected with a strain of *P. gallinaceum* showed a 2.7-fold reduction in oocyst number compared to unirradiated infected counterparts [37], thereby suggesting a potential negative effect of irradiation on mosquito competence for malaria parasites [38, 39]. In contrast, a study on *Anopheles* mosquitoes found that adult gamma-irradiated *An. quadrimaculatus* displayed increased susceptibility to the nematode *Dirofilaria uniformis* [40].

This study aimed to evaluate the effect of a sterilizing dose of gamma-rays from a Caesium-137 source on mosquito competence using the parasite *P. falciparum*, responsible for causing the most severe form of human malaria, and the mosquito *An. arabiensis*, a major vector of *P. falciparum* in Africa. Females of *An. arabiensis* were challenged with sympatric field isolates of *P. falciparum* (14 distinct isolates in total) using direct membrane feeding assays and, through a series of experiments, the effects of irradiation on (i) mosquito competence at two distinct time points over the course of infection (oocyst and sporozoite parasite developmental stages), and (ii) female survival, were examined.

## Methods

### Mosquitoes

Laboratory-reared *An. arabiensis* were obtained from an outbred colony established in 2016 and repeatedly replenished with F1 from wild-caught mosquito females collected in Dioulassoba, a central urban area of Bobo-Dioulasso, south-western Burkina Faso, and identified by routine PCR-RFLP [41]. Mosquitoes were held in 30 × 30 × 30 cm mesh-covered cages under standard insectary conditions (27 ± 2 °C, 70 ± 5% relative humidity, 12:12 LD).

### Irradiation

Irradiation was performed as described in [32]. Prior to irradiation, pupae were transferred from their rearing trays to plastic cups (Ø = 45 mm, h = 85 mm) at similar densities. Cups were randomly assigned to one of two treatment groups: irradiation or unirradiated-control. The pupae density in cups was similar between the two treatment groups and did not exceed 200 pupae per cup. One cm of water was left at the bottom of each cup to limit radiation absorbance by water. Pupae were irradiated at a dose of 95.4 ± 0.9 Gy (mean ± SE) in a Gamma Cell ^137^Cs self-contained gamma source at a rate of 4 Gy/min. In *An. arabiensis* males, this dose induces a high level of sterility [20, 32] while preserving relatively high competitiveness [20]. Cups were placed at the center of the irradiation chamber to maximize dose uniformity within the batch. A dosimetry system was used to measure the accurate dose received by each batch using a Gafchromic® HD-V2 film (Ashland, Bridgewater, NJ, USA) placed on the wall of the cups. After irradiation, the optical density of irradiated films was read at both 458 nm and 590 nm with a dose reader (Dosereader4; Radgen, Budapest, Hungary) and compared to the unirradiated-control. The unirradiated-control group was manipulated in the same way as the irradiated group but was not irradiated. Pupae were then placed in 30 × 30 × 30 cm mesh-covered cages and kept under standard insectary conditions (27 ± 2 °C, 70 ± 10% RH, 12:12 LD) for emergence. Female and male mosquitoes were maintained together on a 5% glucose solution. Between 3 and 6 days after emergence, unirradiated-control and irradiated females were transferred to cardboard cups (Ø = 75 mm, h = 85 mm) at a density of 60 mosquitoes per cup.

### Parasite isolates and mosquito experimental infection

Irradiated and unirradiated-control mosquito females were challenged by using blood drawn from naturally *P. falciparum* gametocyte-infected patients recruited among 5–12-year-old school children in villages surrounding Bobo-Dioulasso, Burkina Faso, using direct membrane feeding assays (DMFA) as previously described [42, 43]. Briefly, thick blood smears were taken from each volunteer, air-dried, Giemsa-stained, and examined by microscopy for the presence of *P. falciparum* at the IRSS laboratory in Bobo-Dioulasso. Asexual trophozoite parasite stages were counted against 200 leucocytes, while infectious gametocytes stages were counted against 1000 leukocytes. Children with asexual parasitemia of > 1000 parasites/µl (estimated based on an average of 8000 leucocytes/ml) were treated in accordance with national guidelines. Asymptomatic *P. falciparum* gametocyte-positive children were recruited for the study.

Gametocyte carrier blood was collected by venipuncture into heparinized tubes. To test for a possible interaction between the natural blocking immunity of the human host [44–46] and the irradiation on mosquito infection, DMFA were performed using either whole donor blood or with replacement of the serum by a non-immune AB serum (see Additional file 1: Text S1). Mosquitoes were starved of glucose solution for 12 h prior to the exposure. Three to six-day-old female mosquitoes, emerged from irradiated or unirradiated-control pupae, were allowed to feed on this blood for 1 h. Non-fed or partially fed females were removed and discarded, while the remaining fully-engorged mosquitoes were maintained in a biosafety room under standard insectary conditions (27 ± 2 °C, 70 ± 10% RH, 12:12 LD). Mosquitoes were provided with a sugar meal consisting in a 5% glucose solution on cotton wool following blood-feeding.

### Experiment 1: Effect of irradiation on *An. arabiensis* competence for *P. falciparum*

Competence was characterized by infection prevalence (i.e. the proportion of mosquitoes that develop infection upon feeding on an infectious blood meal) and intensity (i.e. the average number of parasites among infected mosquitoes). Infection prevalence and intensity were gauged at two distinct points in time over the course of infection (Table 1):

**Table 1:** Summary description of the experiments

Experiment	Time point	Parasite isolates (gam/µl)	Measured traits	Mean ± SE (median) [range] number of mosquitoes for each parasite isolate	
				Irradiated	Unirradiated-control
Experiment 1: Effects of irradiation on *An. arabiensis* competence for *P. falciparum*	7 dpbm	A (64); C (160); D (88); E (32); G (48); H (56); I (48); J (32)	Oocyst prevalence: the number of mosquitoes harboring at least one oocyst in their midguts out of the total number of dissected mosquitoes	47.9 ± 4.9 (50.0) [21–71] (*n* = 383)	47.3 ± 4.9 (51.0) [23–66] (*n* = 378)
			Oocyst intensity: the mean number of oocysts in infected mosquitoes	19.0 ± 2.7 (19.5) [5–31] (*n* = 180)	22.5 ± 3.22 (20.0) [13–37] (*n* = 152)
	14 dpbm	E (32); H (56); I (48); J (32); K (72); L (168); M (32); N (136); O (96); P (96)	Sporozoite prevalence: the number of mosquito head/thorax detected positive to *P. falciparum* using qPCR out of the total number of dissected heads/thoraces	47.3 ± 3.7 (50.0) [17–55] (*n* = 473)	48.9 ± 4.35 (48.5) [27–78] (*n* = 489)
			Sporozoite intensity: the mean number of amplification cycle during qPCR (the lower the Cq, the higher the sporozoite intensity)	25.7 ± 2.7 (26.0) [9–38] (*n* = 257)	24.8 ± 4.7 (23.5) [7–59] (*n* = 248)
Experiment 2: Effects of irradiation on *An. arabiensis* survival	1–7 dpbm	A (64); C (160); D (88); G (48)	From 1 to 7 dpbm, the number and time of death was recorded among mosquitoes exposed to the infectious blood meal	41.3 ± 11.0 (35.5) [22–72] (*n* = 165)	42.0 ± 8.7 (41.5) [24–61] (*n* = 168)
	1–14 dpbm	E (32); H (56); I (48); J (32); K (72); L (168); M (32); N (136); O (96); P (96)	From 1 to 14 dpbm, the number and time of death was recorded among mosquitoes exposed to the infectious blood meal	88.0 ± 9.9 (85.5) [31–146] (*n* = 880)	80.3 ± 11.9 (62.5) [41–137] (*n* = 803)
	1–35 dpbm	J (32)	From 1 dpbm until all mosquitoes had died (35 dpbm), the number and time of death was recorded among both infected mosquitoes and uninfected control mosquitoes	Infected: 26	Infected: 14
				Uninfected: 49	Uninfected: 45

*Abbreviations*: n, total sample size; SE, standard error

(i) On day 7 post-blood meal (dpbm), the midguts of a total of 383 irradiated females and 378 unirradiated-control females fed with blood from one of 8 gametocyte carriers (Table 1) were dissected, stained with 2% mercurochrome, and the presence and number of oocysts (immature, non-transmissible stage of malaria parasites) were recorded using light under the microscopy (400×).

(ii) On 14 dpbm, the heads and thoraces of a total of 473 irradiated and 489 unirradiated-control females fed with blood from one of 10 gametocyte carriers (Table 1) were dissected, and the presence and quantity of sporozoites (mature transmissible stage) were determined using qPCR (see below).

### Experiment 2: Effect of irradiation on *An. arabiensis* survival

Two assays were performed to gauge the effect of irradiation on *An. arabiensis* survival. First, as part of the previous experiments, the survivorship of irradiated and unirradiated-control mosquitoes exposed to each parasite isolate (*n* = 14 isolates) was monitored from 1 to 7 days post-treatment (isolates A, C, D and G) or from 1 to 14 dpbm (isolates E, H, I, J, K, L, M, N, O and P). Every morning at 8:00 h, dead mosquitoes were removed and counted from each cage. The remaining alive mosquitoes used for midgut dissection at 7 and/or 14 dpbm (Experiment 1) were considered in the analysis and given a censoring indicator of “0”.

Secondly, to determine how parasite infection and irradiation interact to influence mosquito longevity, a membrane feeding assay was performed following the same general procedure as described above except that a group of uninfected control mosquitoes was added, and that survival was monitored until all the mosquitoes had died. Uninfected control mosquitoes received heat-treated gametocytic blood to kill the parasite gametocytes as previously described [43, 47, 48]. For each group (irradiated-parasite exposed, irradiated-parasite unexposed, control-parasite exposed and control-parasite unexposed), between 40 and 60 females were placed in one of two 20 × 20 × 20 cm cages to avoid possible cage effect on mosquito survival. Females were fed a 2.5% glucose solution every other day and provided water-soaked cotton *ad libitum*. Dead mosquitoes were counted from each cage (*n* = 8 cages) every morning at 8:00 h and individually stored at -20 °C to determine their infection status using qPCR (see below).

### *Plasmodium falciparum* DNA extraction and qPCR

*Plasmodium falciparum* genomic DNA was extracted from the head-thorax of mosquitoes by grinding tissues with a micro pestle in extraction buffer (0.1 M Tris HCl, pH 8.0, 0.01 M EDTA, 1.4 M NaCl, 2% cetylltrimethyl ammonium bromide). The mixture was incubated at 65 °C for 10 min. Total DNA was extracted with chloroform, precipitated in isopropanol, washed in 70% ethanol, and resuspended in sterile water [49]. Parasite detection was carried out by qPCR, using *P. falciparum* mitochondrial DNA specific primers 5’-TTA CAT CAG GAA TGT TTT GC-3’ and qPCR-PfR 5’-ATA TTG GGA TCT CCT GCA AAT-3’ [50].

### Statistical analyses

All statistical analyses were performed in R (version 3.6.1). Logistic regression by generalized mixed linear models (GLMM, binomial errors, logit link; *lme4* package) were used to test the effect of irradiation on the prevalence of oocysts and sporozoites (Experiment 1), A GLMM with zero truncated negative binomial errors (*glmmTMB* package) was used to test the effect of irradiation on the oocyst intensity (Experiment 1). A GLMM with Gaussian distribution (*lme4* package) was used to test the effect of irradiation on the sporozoite intensity (Cq: mean number of amplification cycle during qPCR, Experiment 1). For each GLMM, the full model included irradiation treatment (irradiated *vs* unirradiated-control) and gametocytemia (the mean number of gametocytes in parasite isolates) as fixed effects and parasite isolate as a random effect. The effect of irradiation on mosquito survivorship (survival assay 1) was analyzed using a Cox’s proportional hazard regression mixed model (*coxme* package) with censoring and with parasite isolate set as a random factor. The effect of irradiation and infection on mosquito survivorship (survival assay 2) was analyzed using Cox’s proportional hazard regression mixed model without censoring and with cage identity set as a random factor. Model simplification used stepwise removal of terms, followed by likelihood ratio tests (LRT). Term removals that significantly reduced explanatory power (*P* < 0.05) were retained in the minimal adequate model.

## Results

### Experiment 1: Effect of irradiation on *An. arabiensis* competence for *P. falciparum*

#### Oocyst prevalence and intensity at day 7 post-blood meal

**Fig. 1:**
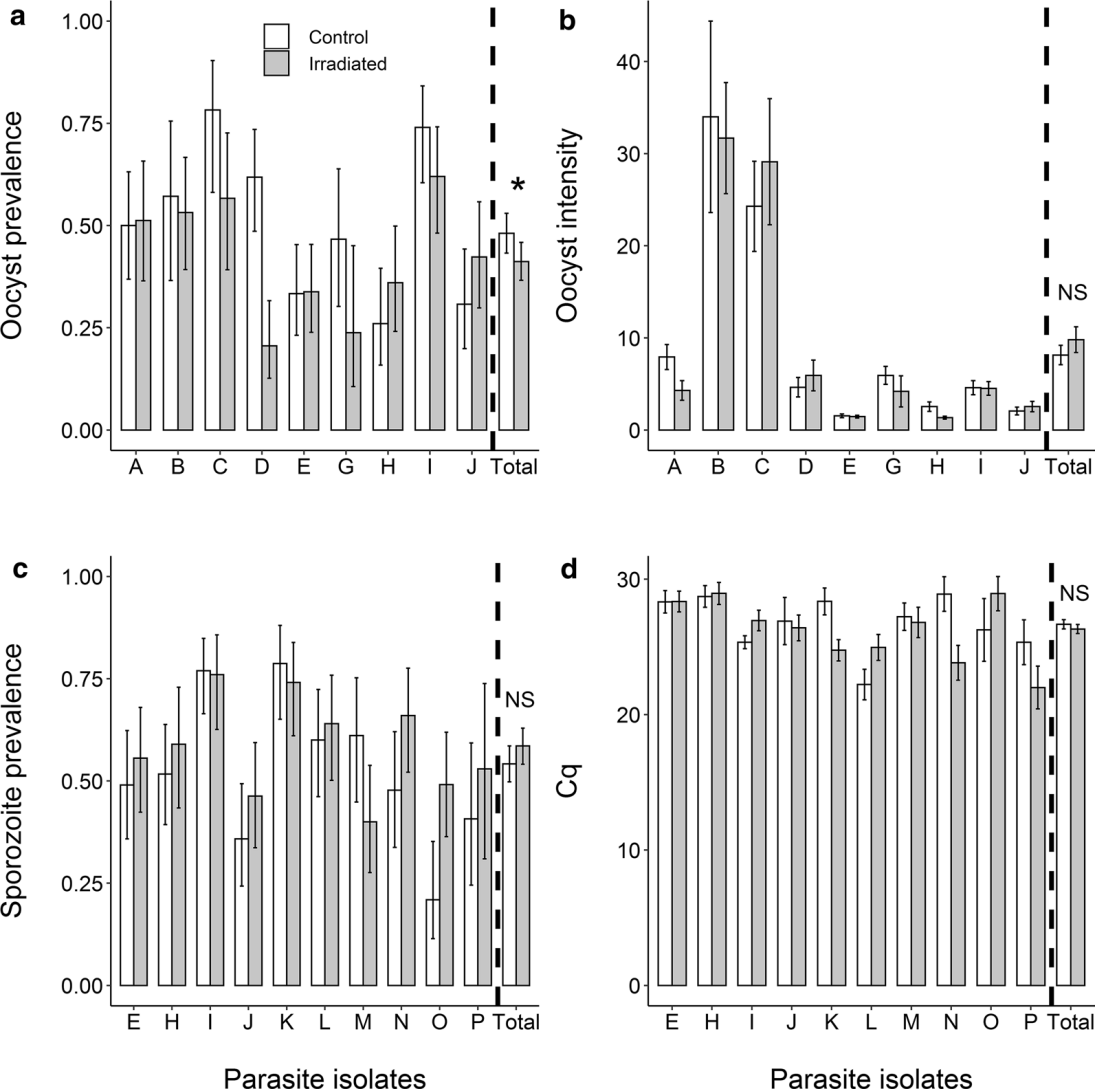
Effect of irradiation on the competence of *Anopheles arabiensis* for natural isolates of *Plasmodium falciparum*. **a** Oocyst prevalence (± 95% CI) on day 7 post-blood meal (dpbm), expressed as the number of mosquito females harboring at least one oocyst in their midguts out of the total number of dissected females, for each treatment (white bars: unirradiated-control mosquitoes, grey bars: irradiated mosquitoes) and for 8 parasite isolates. **b** Infection intensity (± SE) at 7 dpbm, expressed as the mean number of developing oocysts in the guts of infected females, for each treatment and 8 parasite isolates. **c** Sporozoite prevalence (± 95% CI) at 14 dpbm, expressed as the number of mosquito head/thoraces detected positive to *P. falciparum* using qPCR out of the total number of dissected head/thoraces, for each treatment and for 10 parasite isolates. **d** Sporozoite intensity at 14 dpbm, expressed as the mean number (± SE) of amplification cycle during qPCR (the lower the Cq, the higher the sporozoite intensity) for each treatment and for 10 parasite isolates. The asterisk denotes a statistically significant difference (*P*-value < 0.05); NS: not significant

Irradiation reduced the proportion of infected mosquitoes by 16.8% (180 infected unirradiated-control mosquitoes/378 = 47.6%; and 152 infected irradiated mosquitoes/383 = 39.6%; LRT *χ*^2^_1_ = 5.2, *P* = 0.02; Fig. 1a). Although no significant effect of gametocytemia on oocyst prevalence was found (LRT *χ*^2^_1_ = 0.2, *P* = 0.65), there was an interaction between treatment and gametocytemia (LRT *χ*^2^_1_ = 19.5, *P* < 0.001). In particular, while irradiation reduced the mosquito infection rate of parasite isolates C, D, G and I, it had no effect on A and E, and even slightly increased the infection rate of isolates H and J (Fig. 1a).

The mean number of developing oocysts in infected females (i.e. intensity) was not significantly affected by irradiation (LRT *χ*^2^_1_ = 0.0017, *P* = 0.97; Fig. 1b). Gametocytemia had no effect on intensity (LRT *χ*^2^_1_ = 0.54, *P* = 0.46; Fig. 1b). There was a significant interaction between gametocytemia and treatment (LRT *χ*^2^_1_ = 9.58, *P* = 0.002; Fig. 1b) such that irradiation either decreased (isolates A, G and H), increased (C and D) or had no effect (E, I and J) on oocyst intensity.

#### Sporozoite prevalence and intensity at day 14 post-blood meal

The proportion of mosquitoes with disseminated sporozoites in their head/thorax was similar between irradiated and unirradiated-control females (unirradiated-control: 248/489 = 50.7 ± 4%; irradiated: 257/473 = 54.3 ± 5%, LRT *χ*^2^_1_ = 2.56, *P* = 0.11; Fig. 1c). There was no effect of gametocytemia on sporozoite prevalence (LRT *χ*^2^_1_= 0.12, *P* = 0.73; Fig. 1c), and a marginally non-significant interaction between treatment and gametocytemia (LRT *χ*^2^_1_ = 3.5, *P* = 0.06; Fig. 1c).

The mean number of amplification cycles during qPCR (the lower the Cq, the higher the sporozoite intensity) did not vary with irradiation (mean Cq irradiated = 25.57 ± 0.32 (*n* = 257), mean Cq unirradiated-control = 26.02 ± 0.33 (*n* = 248), LRT *χ*^2^_1_ = 0.55, *P* = 0.46; Fig. 1d). Gametocytemia had a significant effect on sporozoite intensity (LRT *χ*^2^_1_ = 7.7, *P* = 0.006), with higher gametocyte density in blood leading to an increase in sporozoite density in mosquito head and thoraces. Finally, there was no interaction between treatment and gametocytemia on sporozoite intensity (LRT *χ*^2^_1_ = 0.04, *P* = 0.85).

### Experiment 2: Effect of irradiation on *An. arabiensis* survival

**Fig. 2:**
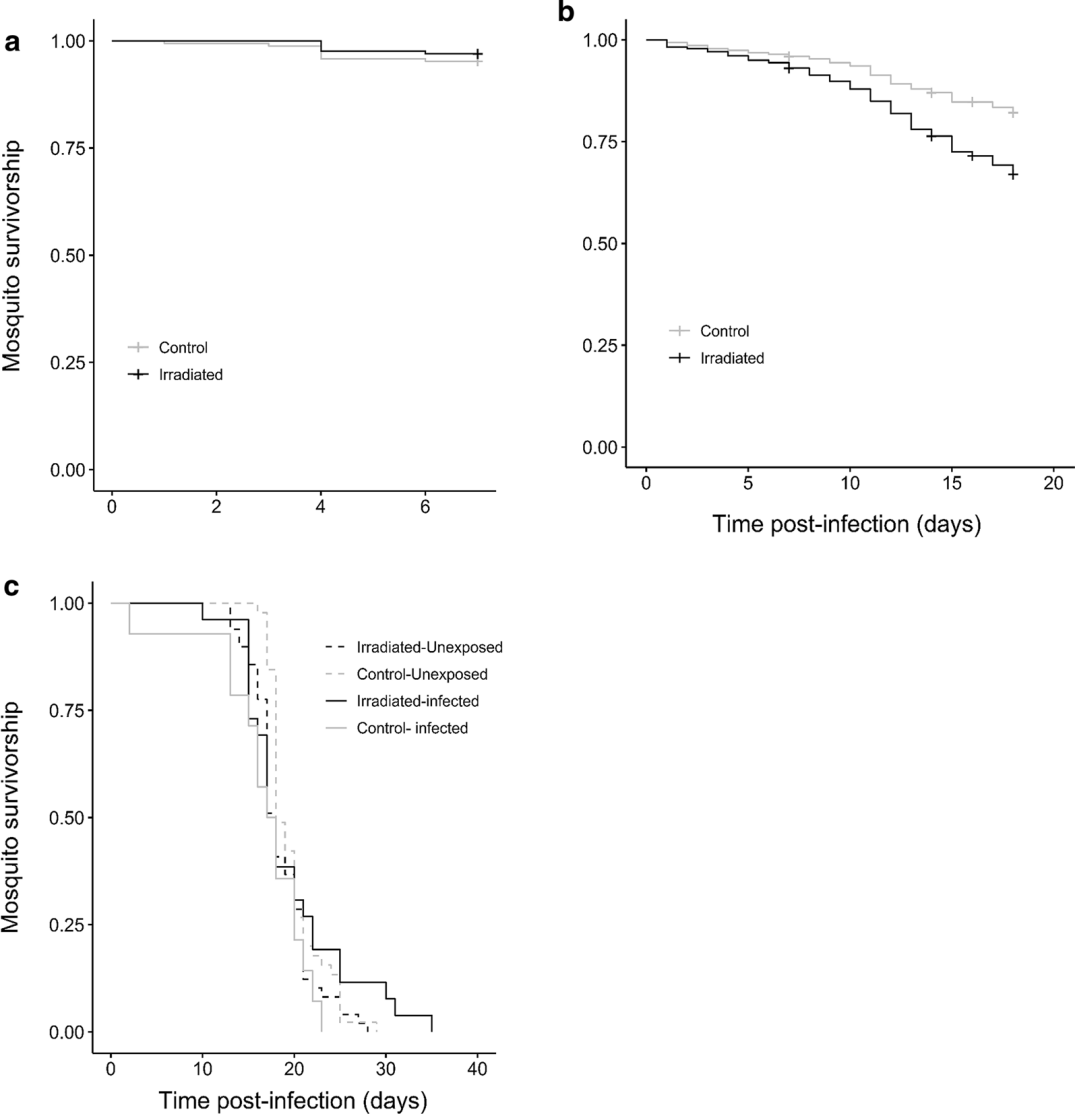
Effect of irradiation on the survival of *Anopheles arabiensis*. **a** Survivorship of malaria-exposed mosquitoes from 1 to 7 dpbm for each treatment (grey line: unirradiated-control, black line: irradiated) using 4 parasite isolates. **b** Survivorship of malaria-infected mosquitoes from 1 to 14 dpbm for each treatment using 10 parasite isolates. **c** Survivorship of both malaria-infected (solid lines) and uninfected unirradiated (dashed lines) mosquitoes from 1 to 35 dpbm for each treatment (grey: unirradiated-control, black: irradiated) using 1 parasite isolate

In the first assay, the survival of females exposed to one of 14 parasite isolates was monitored from 1 to 7 dpbm or from 1 to 14 dpbm (Table 1). The overall survival rate from 1 to 7 dpbm (isolates A, C, D and G) was very high, with 96.1% of mosquitoes (320/333) that survived between 1 to 7 dpbm, and there was no survival difference between irradiated and unirradiated-control mosquitoes (LRT *χ*^2^_1_*=* 1, *P* = 0.31, Fig. 2a). However, from 1 to 14 dpbm (isolates E, H, I, J, K, L, M, N, O and P), irradiated mosquitoes showed a lower survival than unirradiated-control mosquitoes (survival rate irradiated: 79.1% (696/880), unirradiated-control: 88.3% (709/803), LRT *χ*^2^_1_*=* 22.3, *P* < 0.001; Fig. 2b). When focusing on the time range 1–7 dpbm using the 1–14 dpbm dataset, the effect of irradiation on mosquito survival was moderate and marginally non-significant (LRT *χ*^2^_1_*=* 3.67, *P* = 0.055). This suggests that the irradiation-mediated reduction in mosquito survival mostly occurs after 7 dpbm (Fig. 2b).

In the second assay, the survival of irradiated mosquitoes exposed to parasites (*n* = 55), irradiated unexposed (*n* = 49), unirradiated exposed (*n* = 52) and unirradiated unexposed (*n* = 45) females was monitored from 1 to 35 dpbm, when the last mosquito died. The DNA of parasite-exposed dead mosquitoes was extracted to detect the presence of *P. falciparum* using qPCR. Mosquitoes (irradiated or non-irradiated) which remained uninfected upon parasite exposure were excluded from the analysis to focus on the effect of infection and irradiation on mosquito survival. In this second assay using smaller number of mosquitoes (Table 1), there was no effect of irradiation on mosquito survival (LRT *χ*^2^_1_*=* 0.04, *P* = 0.84; Fig. 2c). Infection did not significantly reduce mosquito survival (LRT *χ*^2^_1_*=* 0.05, *P* = 0.82; Fig. 2c). Finally, there was a marginally significant interaction between irradiation and infection (LRT *χ*^2^_1_*=* 4, *P* = 0.045; Fig. 2c), such that irradiation resulted in an increased lifespan in infected mosquitoes but caused a reduced lifespan in uninfected mosquitoes.

## Discussion

Irradiation reduced the proportion of mosquitoes harbouring parasite oocysts upon ingestion of blood meals from gametocyte carriers by 17%, but this effect was highly inconsistent among parasite isolates. Because of this inter-isolate variation and the fact that oocyst and sporozoite data were not collected from exactly the same isolates, the irradiation-mediated reduction in infection observed at 7 dpbm (oocyst stage) was not confirmed at 14 dpbm (sporozoite stages). Indeed, no significant difference in sporozoite prevalence between irradiated and unirradiated control mosquitoes was detected at 14 dpbm. Finally, irradiation either decreased (survival assay 1) or had no effect (assay 2) on the lifespan of *An. arabiensis* females.

Although irradiated females displayed reduced oocyst infection rate compared to non-irradiated individuals, the parasite development was not fully suppressed. If released into the wild, irradiated females will therefore likely contribute to malaria transmission, provided that irradiation does not impair the host-seeking and host-feeding behaviors of these females. Our results therefore highlight the need for perfect sexing tools which would prevent the release of females as part of SIT programmes.

The precise mechanisms behind irradiation-mediated reduction of *Plasmodium* infection at the oocyst stage are not yet clear, but interferences with mosquito immunity, microbiota and/or parasite infectivity mechanisms are likely. Although it is well-known that irradiation causes DNA damage, oxidative stress, and changes in gene expression including immune genes [51], its impact on insect host-pathogen interactions remain generally unclear [52]. While a study found that irradiated Tephritidae flies displayed damaged midgut and peritrophic membranes resulting in decreased bacterial growth [53], irradiated *Spodoptera* butterflies showed increased susceptibility to a nucleopolyhedrosis virus [54]. Similarly, in mosquito-malaria parasite associations, X-ray irradiation caused increased *Ae. aegypti* resistance to *P. gallinaceum* [37, 39], while gamma-ray irradiation enhanced the development of *D. uniformis* in *An. quadrimaculatus* [40]. Together, the few existing studies on this topic suggest that the observed changes in infection level are mediated mostly through radiation damage to the insect midgut rather than through altered immune response such as hemocyte production [52, 55, 56]. In addition, the effects of irradiation on infection seem to be dose-dependent. For example, at a dose of 1000 r of X-ray, the competence of *Ae. aegypti* to *P. gallinaceum* decreased by only 1.15 times compared to unirradiated-control mosquitoes; while at doses between 5000 and 40,000 r, competence decreased by a factor of 2.75 to 4 [37]. Further investigations are required to determine whether the decreased susceptibility of irradiated *An. arabiensis* to *P. falciparum* oocysts is also dose-dependent.

In this study, the effect of irradiation on mosquito infection strongly varied among parasite isolates (Fig. 1). Why irradiation reduced *An. arabiensis* competence for some parasite isolates and not others is unclear. We first postulated that the natural blocking immunity of the human host could play a role. To test this possibility, the natural serum of isolates K to P was replaced by naive AB serum [44–46] (Additional file 1: Figures S1, S2). Similar to assays using unchanged natural serum (isolates A to J), assays with serum replacement showed either increased (L, N, O and P) or decreased (K and M) infection in irradiated mosquitoes (Additional file 1: Figure S2). Here, we used wild parasite isolates from a geographical area characterized by an important genetic diversity [57]. Accordingly, some parasite clones might perform well in irradiated mosquitoes while others would be more infective to non-irradiated mosquitoes. Future genotyping studies of the parasite population used to perform the experimental infections of irradiated mosquitoes would be required to explore this possibility.

The reduction of parasite prevalence observed for the oocyst stage at 7 dpbm (Fig. 1a) was not confirmed for the sporozoite stages at 14 dpmb (Fig. 1c). There are several possible explanations for this uneven pattern. First, and most likely, this was due to the isolate-dependent effect of irradiation (see discussion above), and the fact that the characterization of vector competence for oocyst (Fig. 1a) and sporozoite (Fig. 1c) stages partly relied on different parasite isolates (Table 1). Among the eight isolates used to collect oocyst data (Fig. 1a), half showed a reduced prevalence in the irradiated group (isolates C, D, G and I; Fig. 1a). Among the ten isolates used to collect sporozoite data, the decreased prevalence in irradiated mosquitoes was reported for isolate M and to a lesser extent I and K (Fig. 1c). Secondly, the uneven pattern in Fig. 1a, c could be explained by a differential mortality between irradiated and unirradiated-control mosquitoes exposed to the infectious blood meal. For instance, if infected-irradiated individuals survive better than infected-unirradiated counterparts between 7–14 dpbm, then the relative proportion of infected individuals will increase in the irradiated group only. Our data from the two survival assays (Fig. 2a-c) do not suggest this could be the case. Thirdly, this could be that parasite development is faster in irradiated individuals, thereby increasing the relative proportion of mosquito head/thoraces positive to sporozoites at 14 dpbm. This possibility is supported by the higher proportion of infected mosquitoes with ruptured oocysts (Additional file 2: Figure S3a), the higher ruptured oocyst to intact oocyst ratio (Additional file 2: Figure S3b) and the higher proportion (although not significant) of infected mosquitoes with sporozoites at 14 dpi (Additional file 2: Figure S3c). Exploring the temporal dynamics of *P. falciparum* development using mosquitoes dissected at different time points during the course of infection would provide more detailed and robust information. The number of mosquitoes in our experiments was insufficient to perform such temporal monitoring of the EIP and future experiments are required to confirm our observations at 14 dpi.

The effects of irradiation on the survival of *An. arabiensis* females were inconsistent. In our first assay, the monitoring of 165 irradiated and 168 unirradiated-control females from 1 to 7 dpbm following the ingestion of a gametocyte-infected blood meal revealed no effect of irradiation. Within this period, mosquito survival was very high with only eight deaths in the unirradiated-control group and five in the irradiated group. However, when the monitoring expanded to 14 dpbm on a much larger sample size (880 irradiated and 803 unirradiated-control females), the irradiated group recorded twice as many deaths as the unirradiated-control group (21.25% *vs* 11.71%).

Finally, no significant influence of irradiation was observed as part of the second survival assay in which 26 infected-irradiated, 49 uninfected-irradiated, 14 infected-unirradiated and 45 uninfected-unirradiated mosquitoes were monitored until all individuals had died. Unlike the first assay in which mosquitoes were maintained on a 5% glucose solution *ad libitum*, mosquitoes received a 2.5% glucose solution every other day in this second assay. This was supposed to induce nutritional stress in mosquitoes and help a better detection of the possible effects of radiation on survival [32, 58]. Inconsistent effects of irradiation on the survival of mosquito females were previously observed, with some studies reporting either lifespan reduction [37, 59], no effect [28, 59–61] or even an increase [59]. For example, in the mosquito *Ae. polynesiensis*, irradiation of females < 24 h post-pupation at 20 Gy and 40 Gy induced a significant lifespan reduction compared to non-irradiated females, while irradiation at 30 Gy had no effect and irradiation at 40 Gy of females > 24 h post-pupation boosted female lifespan. If confirmed in field conditions, the irradiation-mediated reduction of mosquito lifespan observed from 1 to 14 dpbm would not be strong enough to prevent the completion of the *Plasmodium* incubation period and hence the contribution of these females to malaria transmission [59].

## Conclusions

Our data indicate that irradiation of female *An. arabiensis* can reduce competence and survival, but not to the point of preventing malaria transmission. Irradiated females therefore remain potential vectors and further studies are required to develop fully effective sexing tools to prevent possible release of irradiated females into the wild. Until we find such sexing tools, it will be important to expand our knowledge on the radiation biology of female mosquito vectors.

## Supplementary information

**Additional file 1: Text S1.** Serum replacement assay. **Figure S1.** Relationship between sporozoite prevalence and gametocytemia (number of gametocytes/µl of blood) (**a**) and between Cq and gametocytemia (**b**). Blue lines indicate isolates for which the plasma was kept unchanged and black lines indicate isolates with naïve AB serum. **Figure S2.** Effect of irradiation on sporozoite prevalence (± 95 % CI) at 14 dpbm (**a**), expressed as the number of mosquito head/thoraces detected positive to *Plasmodium falciparum* using qPCR out of the total number of dissected head/thoraces, for each treatment and for 10 parasite isolates; and on sporozoite intensity at 14 dpbm (**b**), expressed as the mean number (± SE) of amplification cycle during qPCR (the lower the Cq, the higher the sporozoite intensity) for each treatment and for 10 parasite isolates. NS: not significant.

**Additional file 2**: **Text S2.** Oocyst rupture assay. **Figure S3.** Effect of irradiation on *P. falciparum* oocyst rupture in mosquito guts and sporozoite dissemination in head/thoraces on day 14 post-infection for 6 parasite isolates. **a** Proportion of infected mosquitoes with ruptured oocysts (± 95% CI), expressed as the number of mosquitoes with at least one ruptured oocyst out of the total number of oocyst-infected mosquitoes for each treatment (white bars: control mosquitoes; grey bars: irradiated mosquitoes). **b** Proportion of ruptured oocysts (± 95% CI), expressed as the number of ruptured oocysts out of the total number of oocysts (intact + ruptured) for each treatment. **c** Proportion of oocyst-infected mosquitoes with sporozoites in their head and thorax (± 95% CI), for each treatment. *P < 0.05; ***P < 0.001; NS: not significant. **Figure S4.** Immature developing oocysts. **Figure S5.** Mature and immature oocysts. **Figure S6.** Ruptured and unruptured mature oocysts. **Figure S7.** Ruptured oocysts. **Table S1.** Summary description of the experiments.

## Data Availability

Data supporting the conclusions of this article are included within the article and its additional files. The raw datasets are available from the corresponding author.

## Abbreviations

dpbm: days post-blood meal
SIT: sterile insect technique
L:D: light dark
qPCR: real-time polymerase chain reaction
DMFA: direct membrane feeding assay
SE: standard error
