# Supplementary material for: Effect of irradiation on the survival and susceptibility of female Anopheles arabiensis to natural isolates of Plasmodium falciparum
Source: Parasit Vectors. 2020 May 20;13:266. doi: 10.1186/s13071-020-04135-w (PMC7238563; doi:10.1186/s13071-020-04135-w)
Supplement: Supplementary file 2 — Additional file 2: Text S2. Oocyst rupture assay. Figure S3. Effect of irradiation on P. falciparum oocyst rupture in mosquito guts and sporozoite dissemination in head/thoraces on day 14 post-infection for 6 parasite isolates. a Proportion of infected mosquitoes with ruptured oocysts (± 95% CI), expressed as the number of mosquitoes with at least one ruptured oocyst out of the total number of oocyst-infected mosquitoes for each treatment (white bars: control mosquitoes; grey bars: irradiated mosquitoes). b Proportion of ruptured oocysts (± 95% CI), expressed as the number of ruptured oocysts out of the total number of oocysts (intact + ruptured) for each treatment. c Proportion of oocyst-infected mosquitoes with sporozoites in their head and thorax (± 95% CI), for each treatment. *P < 0.05; ***P < 0.001; NS: not significant. Figure S4. Immature developing oocysts. Figure S5. Mature and immature oocysts. Figure S6. Ruptured and unruptured mature oocysts. Figure S7. Ruptured oocysts. Table S1. Summary description of the experiments. [file 13071_2020_4135_MOESM2_ESM.docx]

**Additional file 2**

**Text S2. Oocyst rupture assay**

On 14 dpbm, 276 irradiated and 243 control mosquito females fed an infectious blood from one of 6 gametocyte carriers were dissected for the microscopic observation of oocysts (see figures 4 to 7 below) in midguts and the qPCR detection of sporozoites in head/thoraces (Table S1 below). Oocyst rupture in mosquito midgut and sporozoite invasion of salivary glands is highly asynchronous: while some oocysts are intact and keep developing on 14 dpbm, others have already ruptured and released their sporozoites. To explore possible difference in the timing of sporozoite dissemination in mosquito salivary glands between irradiated and control females, three traits were measured:

1. the proportion of infected mosquitoes with ruptured oocysts on 14 dpbm. This is the number of mosquitoes with at least one ruptured oocyst (figure 7) in their midguts at 14 dpbm out of the total number of infected mosquitoes (i.e. harboring either intact and/or ruptured oocysts (figure 6);
2. the proportion of ruptured oocysts (figure 7) at 14 dpbm. This is, for each infected mosquito, the number of ruptured oocysts out of the total number of oocysts (intact + ruptured (figure 6));
3. the proportion of oocyst-infected mosquitoes with sporozoites in their head and thorax at 14 dpbm. This is the number of oocyst-infected mosquitoes harboring sporozoites in their head/thoraces at 14 dpbm out of the total number of infected mosquitoes (i.e. harboring either intact and/or ruptured oocysts ().

**Statistical analyses:** All statistical analyses were performed in R (version 3.6.1). Logistic regression by generalized mixed linear models (GLMM, binomial errors, logit link; lme4 package) were used to test the effect of irradiation on (i) the proportion of infected mosquitoes with ruptured oocysts, (ii) the proportion of ruptured oocysts, (iii) the proportion of oocyst-infected mosquitoes with sporozoites in their head and thorax. For each GLMM, the full model included irradiation treatment (irradiated vs. unirradiated-control) and gametocytemia (the mean number of gametocytes in parasite isolates) as fixed effects and parasite isolate as a random effect. Model simplification used stepwise removal of terms, followed by likelihood ratio tests (LRT). Term removals that significantly reduced explanatory power (P < 0.05) were retained in the minimal adequate model

**Results**

Uninfected mosquitoes were excluded from the analysis and the parasite oocyst rupture in mosquito guts and sporozoite dissemination to head/thoraces were compared between irradiated and control infected individuals (N irradiated = 144/276 (52 %), N control = 124/243 (51 %)). Among these infected mosquitoes, the proportion of individuals with at least one ruptured oocyst in their midgut at 14 dpbm was higher in irradiated females than in control counterparts (*LRT X^2^_1_* = 5.8, P = 0.016, Figure S3a, below). In particular, 86 % (124/144) of irradiated infected mosquitoes had at least one ruptured oocyst in their midguts, while only 75 % (93/124) of control-infected females exhibited ruptured oocysts. This result suggests that the release of sporozoites from oocysts happened earlier in irradiated than in control females.

In addition, the proportion of ruptured oocysts was higher in irradiated mosquitoes (irradiated: 1509 ruptured oocysts out of a total of 1817 counted oocysts (83 %), controls: 1443 ruptured oocysts out of a total of 2068 counted oocysts (69.8 %), *LRT X^2^_1_* = 85, P < 0.001, Figure S3b, below), further suggesting that irradiation speeded up oocyst maturation and sporozoite release.

Finally, the proportion of oocyst-infected mosquitoes with disseminated sporozoites in their head/thorax was not affected by irradiation treatment (*LRT X^2^_1_* = 2, P = 0.12, Figure S3c, below). There was no main effect of gametocytemia on the proportion of oocyst-infected mosquitoes with disseminated sporozoites in their head/thorax (*LRT X^2^_1_* = 1.65, P = 0.2). There was a significant interaction between gametocytemia and treatment (*LRT X^2^_1_* = 4.6, P = 0.03), with irradiation either decreasing (isolates M), or increasing (K, N, O, P) the proportion of oocyst-infected mosquitoes with disseminated sporozoites.


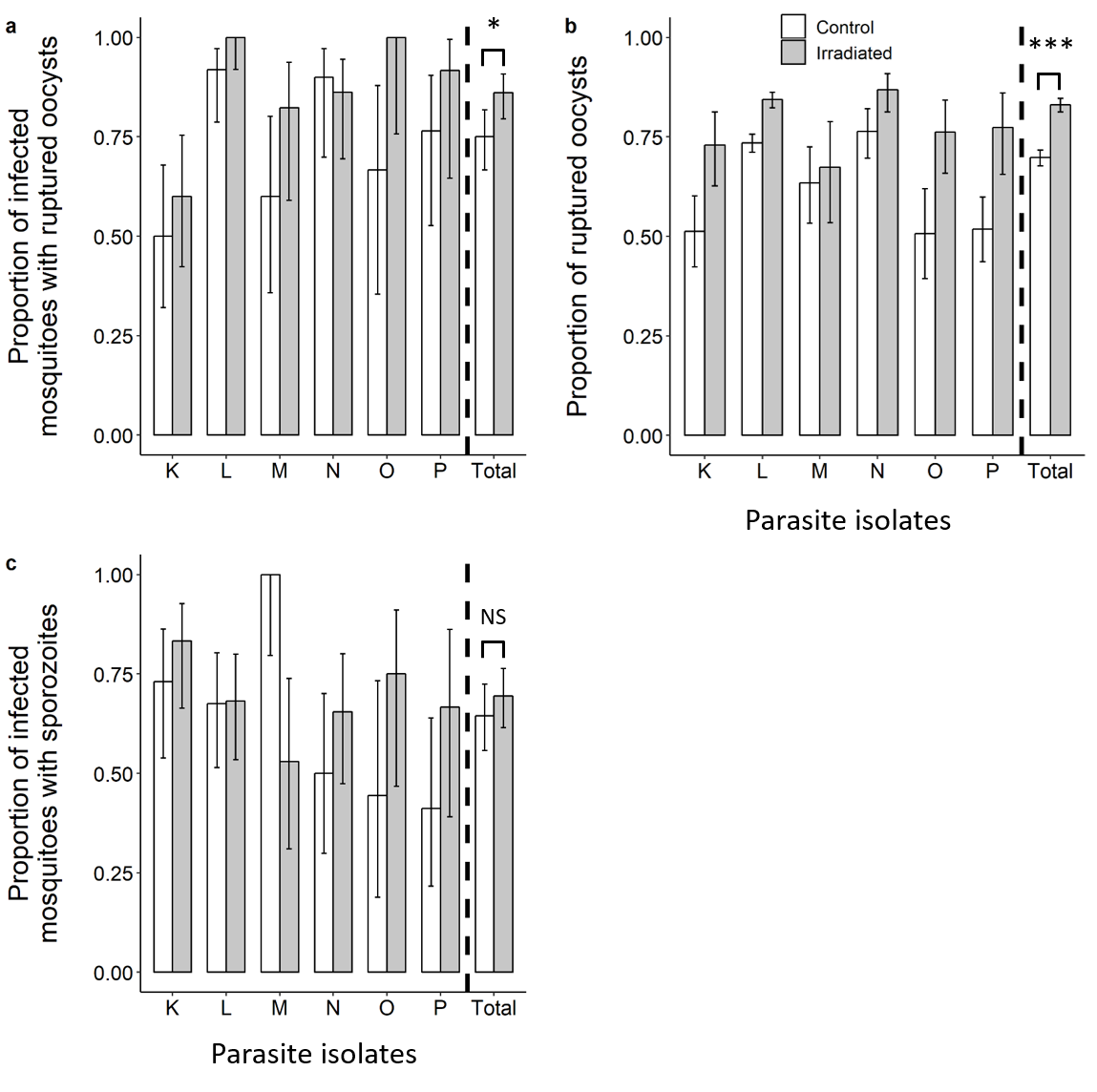


**Figure S3: Effect of irradiation on *P. falciparum* oocyst rupture in mosquito guts and sporozoite dissemination in head/thoraces on day 14 post-infection**. (a) Proportion of infected mosquitoes with ruptured oocysts (± 95% CI), expressed as the number of mosquitoes with at least one ruptured oocyst out of the total number of oocyst-infected mosquitoes (i.e. harboring either intact and/or ruptured oocysts) on day 14 post-infection for each treatment (white bars: control mosquitoes, grey bars: irradiated mosquitoes) and for 6 parasite isolates. (b) Proportion of ruptured oocysts (± 95% CI), expressed as the number of ruptured oocysts out of the total number of oocysts (intact + ruptured) (i.e. the ruptured oocysts - to - intact oocysts ratio) on day 14 post-infection for each treatment and 6 parasite isolates. (c) Proportion of oocyst-infected mosquitoes with sporozoites in their head and thorax (± 95% CI), expressed as the number of oocyst-infected mosquitoes harboring sporozoites in their head/thoraces out of the total number of infected mosquitoes on day 14 post-infection**,** for each treatment and for 6 parasite isolates. The asterisk denotes a statistically significant difference (*P*-value < 0.05); NS: not significant.

**Discussion**

Our results suggest an earlier sporozoite invasion of salivary glands among irradiated females. This is supported by the higher proportion of infected mosquitoes with ruptured oocysts (Additional file 2, Figure S3a), the higher proportion of ruptured oocysts (Additional file 2, Figure S3b), and the higher proportion (although not significant) of infected mosquitoes with sporozoites at 14 dpbm (Additional file 2, Figure S3c). Gamma-irradiation might speed up *Plasmodium* development within *Anopheles* vectors. Shorter parasite’s Extrinsic Incubation Period (EIP) following insect host irradiation was previously described in *Trypanosoma spp* – infected tsetse flies [1]. In this system, the parasite migration to the haemocoel occurred earlier in irradiated than in unirradiated-control flies, possibly because of changes in the ultrastructural organization of the insect gut [2]. Exploring the temporal dynamics of *P. falciparum* development using mosquitoes dissected at different time points during the course of infection would provide more detailed and robust information. The number of mosquitoes in our experiments was insufficient to perform such temporal monitoring of the EIP and future experiments are required to confirm our observations made at 14 dpbm.

**References**

1. Moloo SK. Cyclical Transmission of Pathogenic Trypanosoma Species by Gamma-Irradiatéd Sterile Male *Glossina Morsitans Morsitans*. Parasitology. 1982;

2. Stiles JK, Molyneux DH, Wallbanks KR, der Vloedt AMV Van. Effects of γ Irradiation on the Midgut Ultrastructure of *Glossina palpalis* Subspecies Effects of g Irradiation on the Midgut Ultrastructure of *Glossina palpalis* Subspecies. Radiat Res. 1989;

**Table S1: Summary description of the experiments**

| **Experiment** | **Time point** | **Parasites isolates (gam/µl)** | **Measured traits** | **Total sample size (N total)** | |
| --- | --- | --- | --- | --- | --- |
|  |  |  |  | **Mean ± SE (median) [range] number of mosquitoes for each parasite isolate** | |
|  |  |  |  | **irradiated** | **unirradiated-control** |
| **Experiment 2**: Effects of irradiation on *P. falciparum* oocyst rupture in mosquito guts and sporozoite dissemination in head/thoraces | 14 dpbm | K (72), L (168), M (32), N (136), O (96), P (96) | **Proportion of infected mosquitoes with ruptured oocysts**: the number of mosquitoes with at least one ruptured oocyst out of the total number of infected mosquitoes (i.e. harboring either intact and/or ruptured oocysts) | N total = 276 | N total = 243 |
|  |  |  | **Proportion of ruptured oocysts**: the number of ruptured oocysts out of the total number of oocysts (intact + ruptured) | 24 ± 5  (23) [12-44] | 20.7 ± 4  (18.5) [9-37] |
|  |  |  | **Proportion of oocyst-infected mosquitoes with sporozoites in their head and thorax**: the number of oocyst-infected mosquitoes harboring sporozoites in their head/thoraces out of the total number of infected mosquitoes |  |  |


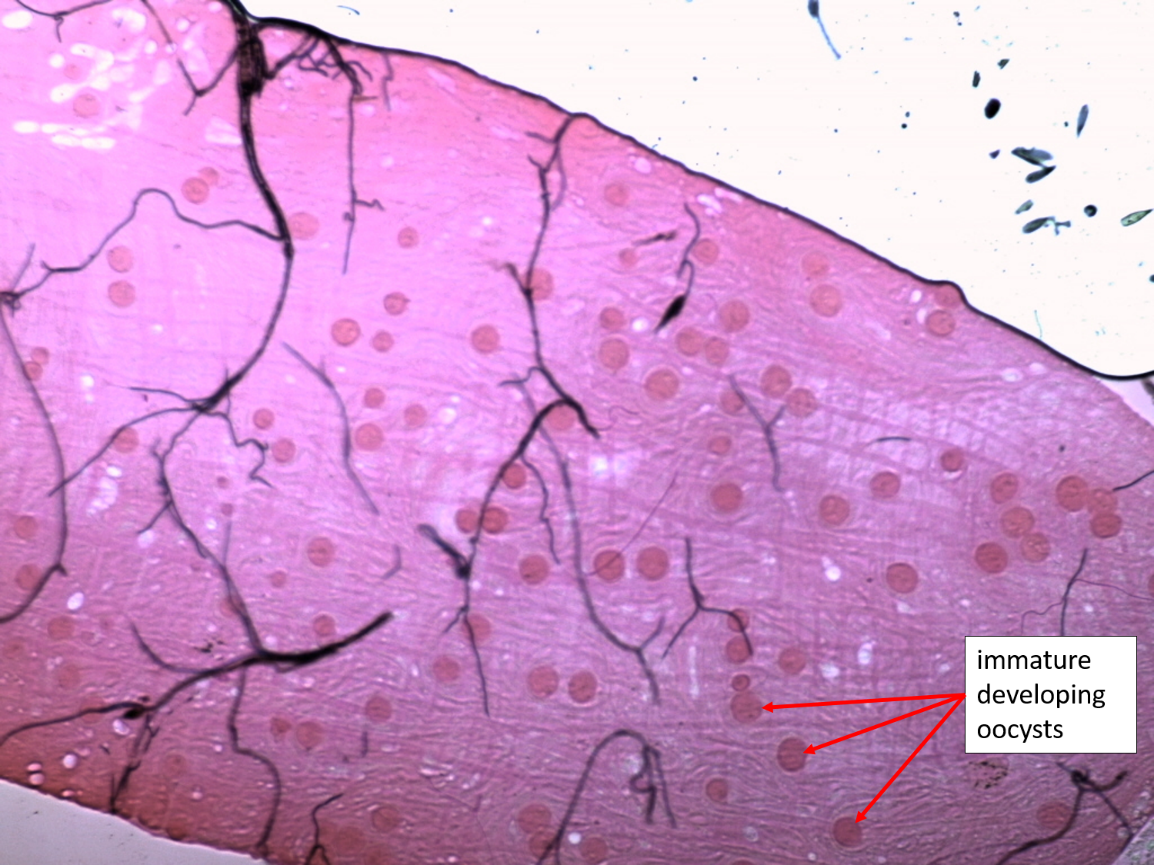


**Figure S4:** Microscopic observation (x100) of a mosquito gut dissected at 7 days post-blood-meal (dpbm) and harboring dozens of immature developing oocysts. Mosquitoes were maintained at 27°C during parasite development. Credit: Guissou Edwige


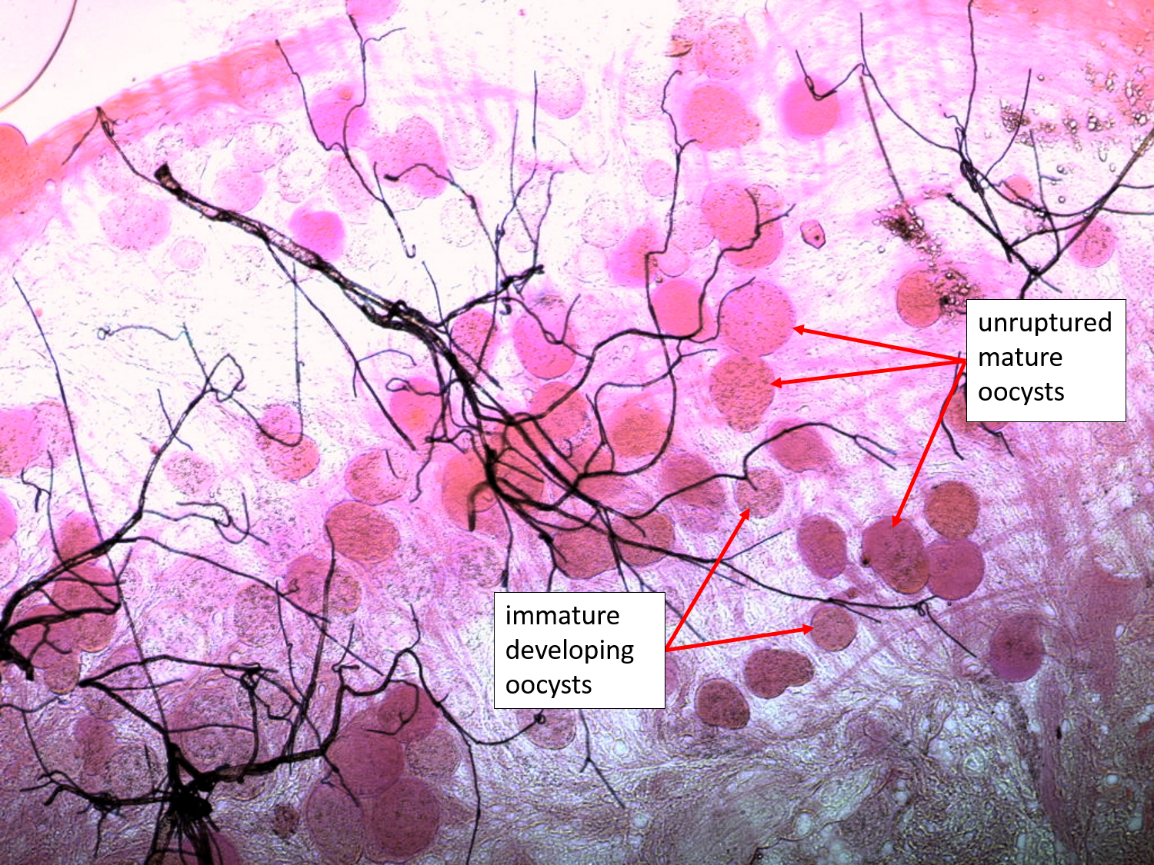


**Figure S5:** Microscopic observation (x 200) of a mosquito gut dissected at 10 days post-blood-meal (dpbm) and harboring both mature and immature oocysts. Note the protrusion of the capsule of some oocysts that may here result from the application of the coverslip. In our experiment, these distorted oocysts were recorded as intact unruptured oocysts because the capsule has not broken yet. Mosquitoes were maintained at 27°C during parasite development. Credit: Guissou Edwige


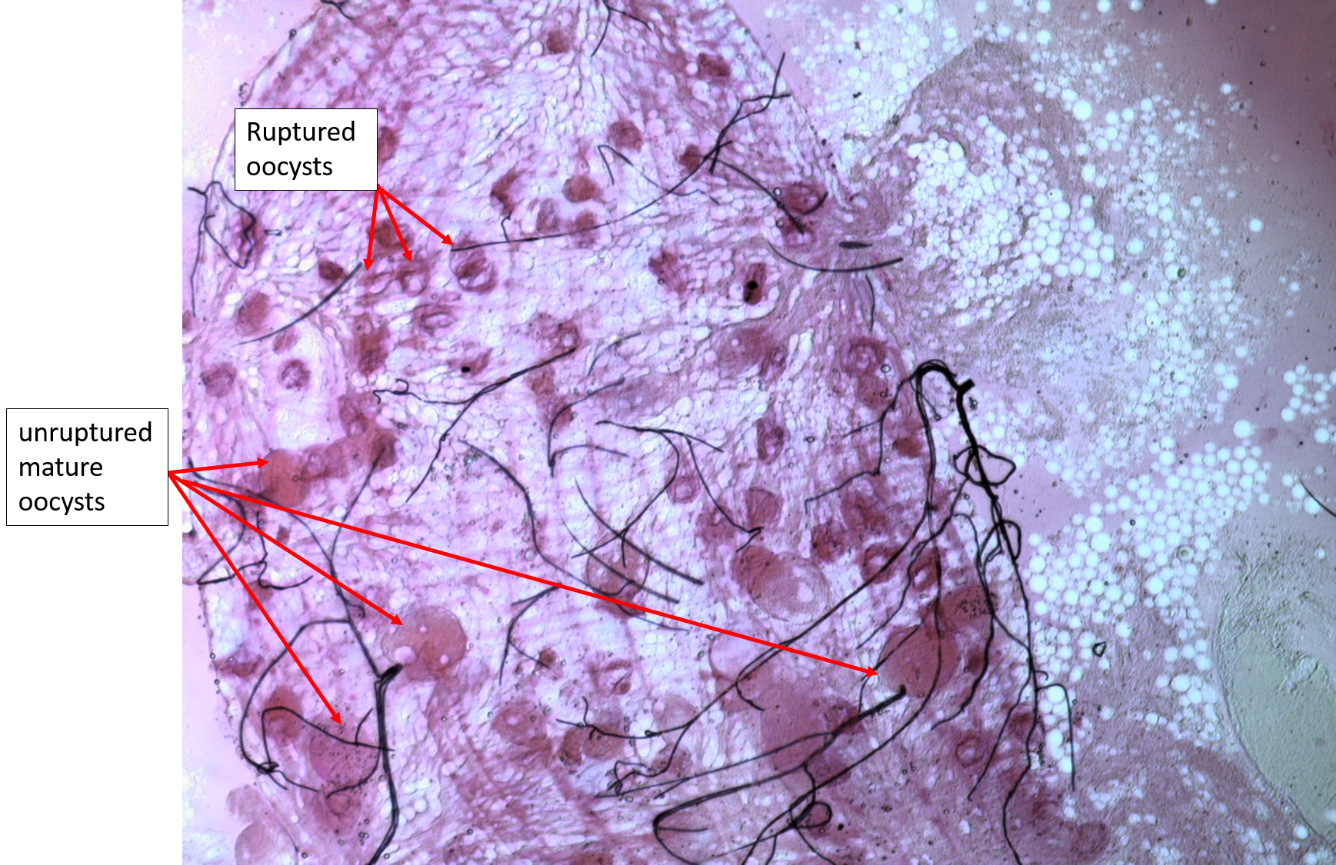


**Figure S6:** Microscopic observation (x 100) of a mosquito gut dissected at 14 days post-blood-meal (dpbm) and harboring both ruptured and unruptured mature oocysts. The ruptured oocysts are characterized by empty and withered capsules. Ruptured oocysts look like a hatched egg from which only the shell remains. Mosquitoes were maintained at 27°C during parasite development. Credit: Guissou Edwige


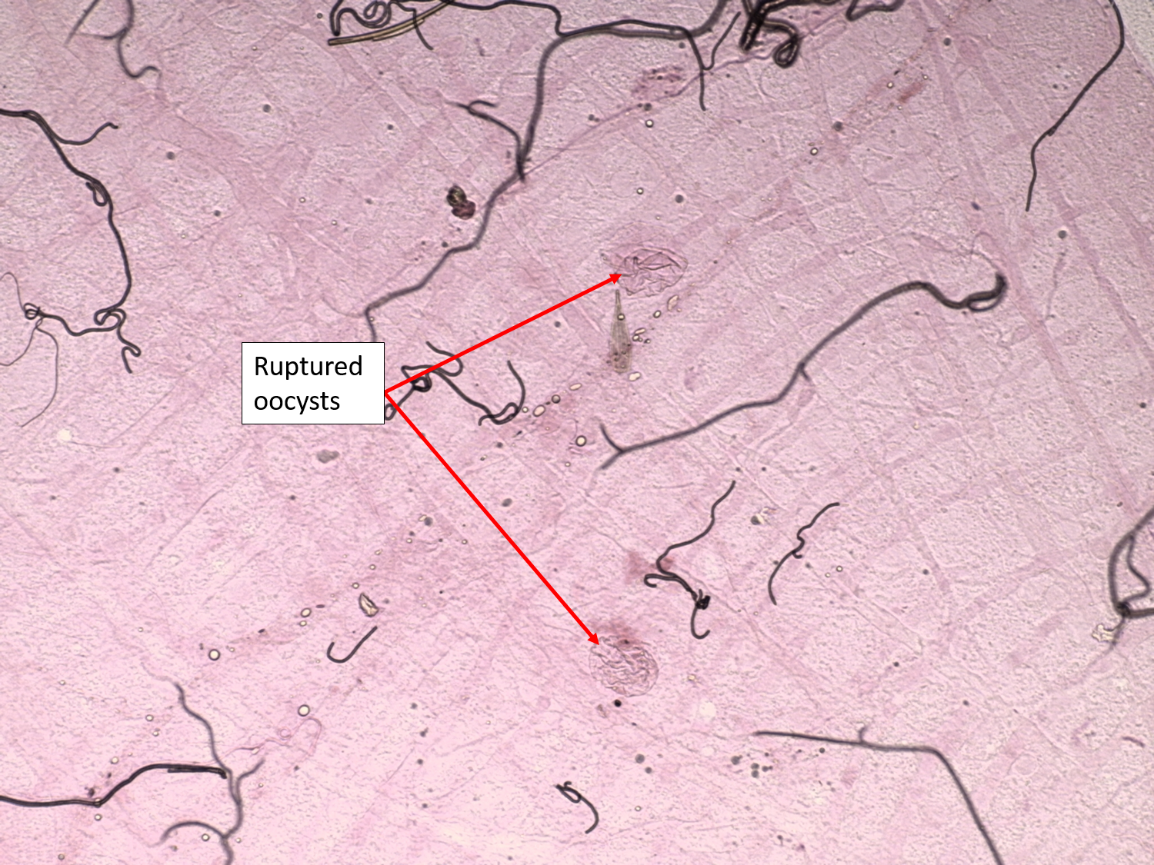


Figure S7: Microscopic observation (x 200) of a mosquito gut dissected at 14 days post-blood-meal (dpbm) and harboring ruptured oocysts only. The ruptured oocysts are characterized by empty and withered capsules. Ruptured oocysts look like a hatched egg from which only the shell remains. Mosquitoes were maintained at 27°C during parasite development. Credit: Guissou Edwige
